# Advancing environmental health sciences through implementation science

**DOI:** 10.1186/s12940-022-00933-0

**Published:** 2022-12-23

**Authors:** Gila Neta, Lindsey Martin, Gwen Collman

**Affiliations:** 1 Division of Cancer Control and Population Sciences, National Cancer Institute, National Institutes of Health, 9609 Medical Center Drive, Rockville, MD 20850 USA; 2 Division of Extramural Research and Training, National Institute of Environmental Health Sciences, National Institutes of Health, Research Triangle Park, NC, 27709 USA; 3 Office of Scientific Coordination, Planning and Evaluation (SCOPE), National Institute of Environmental Health Sciences, National Institute of Health, Research Triangle Park, NC, 27709 USA

**Keywords:** Environmental health sciences, Implementation science, Translation, Environmental health

## Abstract

**Background:**

Environmental health sciences have identified and characterized a range of environmental exposures and their associated risk for disease, as well as informed the development of interventions, including recommendations, guidelines, and policies for mitigating exposure. However, these interventions only serve to mitigate exposures and prevent disease if they are effectively disseminated, adopted, implemented, and sustained.

**Main body:**

Numerous studies have documented the enormous time lag between research and practice, noting that dissemination and implementation are not passive processes but rely on active and intentional strategies. Implementation science seeks to build the knowledge base for understanding strategies to effectively disseminate and implement evidence and evidence-based interventions, and thus, bridge the research-to-practice gap.

**Conclusion:**

Environmental health researchers are well positioned to advance health promotion and disease prevention by incorporating implementation science into their work. This article describes the rationale for and key components of implementation science and articulates opportunities to build upon existing efforts to advance environmental health supported by the National Institute of Environmental Health Sciences and National Institutes of Health broadly.

## Background

The National Institute of Environmental Health Sciences (NIEHS) seeks to understand “how the environment affects people in order to promote healthier lives.” NIEHS has invested substantially in environmental health research which has enumerated scores of chemicals that may cause disease, elucidated molecular mechanisms for disease initiation and progression, and informed the development of interventions, including recommendations, guidelines, and policies for mitigating exposure. However, these evidence-based interventions, recommendations, guidelines, and policies (hereafter referred to collectively as “interventions”) are only effective at mitigating exposures and preventing disease if they are effectively disseminated, adopted, implemented, and sustained.

Numerous studies have documented the enormous time lag between clinical research and practice. While in healthcare this estimate has hovered around 17 years [1, 2], the time lag can be substantially longer for environmental health evidence to result in changes to policy and practice. For example, we continue to see high rates of smoking despite the 1964 Surgeon General Report and effective tobacco control interventions including those which reduce exposure to secondhand smoke in the workplace. Air pollution is another example. Despite significant documentation of mortality associated with air pollution as early as the 1950s [3], the Clean Air Act wasn’t established until 1970 and air pollution continues to plague many U.S. cities. Even longer lags have been seen with arsenic, which we’ve known is carcinogenic for over a century, yet arsenic levels in the U.S. population continue to be above the U.S. Federal Government’s national health objectives goal as outlined in Healthy People 2020. Other examples include lead and mercury, for which it took decades for the evidence on their health effects to translate to global policy action [4], and yet millions of children continue to be exposed to high levels of these toxins.

Increasingly researchers have come to appreciate that dissemination and implementation of evidence-based interventions are not passive processes but rely on active and intentional strategies that should be informed by theories, stakeholders, and evidence. Implementation science seeks to build the knowledge base for understanding strategies to effectively disseminate, implement, and sustain evidence and evidence-based interventions, and thus, bridge the research-to-practice gap [5, 6]. Given the complex influences and global nature of environmental exposures, environmental health researchers are well positioned to advance disease prevention by integrating implementation science into their work.

In this article, we describe the key components of implementation science and articulate ways that environmental health researchers can build upon existing efforts to advance environmental health through implementation science. NIEHS emphasizes the importance of engaging with affected communities, practitioners, policymakers, and other partners across multiple sectors, and the need to develop and equitably implement effective, evidence-based environmental health interventions to prevent and mitigate harmful exposures and reduce environmental health disparities. Table 1 provides a roadmap for environmental health scientists to consider how implementation science could advance their work in alignment with NIEHS goals. Further, we provide an example of a network of environmental health researchers and implementation scientists collaborating to advance the implementation of clean cookstove interventions to reduce household air pollution and improve population health. We hope this example may serve to drive future research directions and collaborations.

**Table 1:** Implementation Science and NIEHS Research Priorities

Priority Area	Integration of Implementation Science
**Evidence-Based Prevention and Intervention:** moving evidence into action through the development and testing of interventions that can improve human health by preventing or reducing harmful environmental exposures	• Incorporation of implementation science into intervention development using effectiveness-implementation hybrid designs (e.g., ‘designing for implementation’ by collecting data on barriers and facilitators that may impact intervention adoption and sustained use) • Identification and testing of implementation strategies to facilitate the effective uptake of environmental health interventions to maximize public health impact • Example: the Community Mobilization for Improved Clean Cookstove Uptake, Household Air Pollution Reduction, and Hypertension Prevention study funded through the National Heart, Lung & Blood Institute uses a hybrid design to test both effectiveness (if the clean cooking technology improves blood pressure) and implementation (testing a strategy to improve adoption of the stoves) (NCT05048147)
**Environmental Health Disparities and Environmental Justice:** understanding and addressing the disparate health impacts of environmental exposures on populations with health disparities (https://www.nimhd.nih.gov/about/overview/), including the intersection of social and structural determinants of health (e.g., race, income) with environmental exposures	• Examining multi-level (individual, community, organizational, structural) factors, including social determinants of health (SDOH), that could influence equitable implementation of environmental health interventions [7] • Understanding contextual factors that influence equitable implementation to design and deliver interventions that will mitigate and not exacerbate existing environmental health disparities • Example: Clean Cooking Implementation Science Network projects highlight contextual barriers and facilitators to liquid petroleum gas stove adoption and how barriers such as cost or lack of transportation limit scale-up and spread of an effective intervention [8, 9]
**Emerging Environmental Health Issues:** building resilience in the face of emerging environmental threats including human and natural caused environmental disasters, including acute and long-term impacts of climate change on human health	• Understanding how to effectively adapt interventions and policies in the face of environmental disasters and rapidly changing evidence • Use of rapid cycle implementation research designs (e.g., approaches that allow for incremental and contextually informed modifications) [10] • Hypothetical Example: Designing implementation strategies that are more anticipatory or proactive vs. reactive in the face of environmental disasters, to facilitate and enhance preparedness.
**Community and Multi-Sectoral Partnerships:** building and sustaining partnerships with communities impacted by environmental exposures and across multiple organizations and sectors (federal, state, tribal, public health agencies)	• Equitable engagement of communities to ensure community concerns and priorities are incorporated into intervention development and choice of implementation strategies [11] • Developing multi-sectoral partnerships to facilitate the scale-up and spread of environmental health interventions and assure sustainability. • Engaging policy makers to support the implementation of environmental health interventions • Example: Collaborating with community working groups to co-design clean solutions to plastic waste disposal and reduce plastic waste incineration

### What is implementation science?

Implementation science is the study of methods to promote the adoption and integration of evidence and evidence-based practices, interventions and policies into routine healthcare and community settings to improve health [5, 6]. The National Institutes of Health (NIH) issued funding announcements in 2005 to support research on understanding barriers to dissemination and implementation and develop and test strategies to overcome those barriers.

For the purposes of the funding announcements, the NIH makes a distinction between dissemination research and implementation research. Dissemination research is defined as the study of the “targeted distribution of information and intervention materials to a specific public health, clinical practice, or policy audience” [5]. The intent is to understand how, when, by whom, and under what circumstances evidence and the associated evidence-based interventions can be most effectively communicated and integrated into practice. It accounts for all the stages of dissemination, including the creation, packaging, transmission, and reception of the knowledge and associated interventions. These are steps we often take for granted but that can impact the effectiveness of our dissemination efforts.

Implementation research has been defined as the study of the use of strategies to adopt and integrate evidence-based health interventions into clinical and community settings to improve individual outcomes and benefit population health [5]. While clinical trials test the effectiveness of interventions to improve health outcomes, implementation research focuses on understanding *how* those interventions can best be delivered to ensure they have the intended impact on health. Implementation studies develop and test strategies to ensure effective implementation. Rather than focusing on individual health outcomes, implementation studies focus on proximal outcome measures that demonstrate implementation success, such as measures of acceptability, adoption, appropriateness, costs, feasibility, fidelity, penetration, and sustainability [12]. The goal is to identify a strategy or set of strategies that will maximize effective adoption, implementation and sustainability of an evidence-based intervention, and thus, ultimately improve population health. Additional key components of implementation science are described in the following paragraphs with a guiding example from environmental health on the adoption and use of clean cookstoves to reduce household air pollution. This example comes from the Clean Cooking Implementation Science Network, which was established and funded by the National Institutes of Health in partnership with the Environmental Protection Agency (EPA), Centers for Disease Control and Prevention (CDC), U.S. Agency for International Development (USAID), and the Clean Cooking Alliance. The network consists of environmental health researchers and implementation scientists studying strategies to promote the adoption, use, and scale-up of clean cooking technologies around the globe.

#### Theories, frameworks, and models

The field of implementation science hinges on theories, frameworks, and models to inform dissemination and implementation processes and help determine the most effective strategies to overcome barriers to dissemination and implementation. These theories, frameworks, and models (hereafter referred to collectively as frameworks) typically recognize the importance of context as well as the multiple levels of influence on dissemination and implementation processes. More than 60 frameworks are used in the field [13] for a variety of purposes including to inform processes and determinants of implementation, as well as to evaluate implementation success [14].

Some of the most commonly used frameworks in NIH-funded studies include Everett Rogers’ Diffusion of Innovations [15] and the Consolidated Framework for Implementation Research [16], both of which posit that the decision to adopt and successfully implement an intervention is influenced not only by the characteristics of the intervention itself but also by the setting or context in which that intervention is implemented. Further, these frameworks recognize the multiple levels of influence in a given context, from the organizational or community setting to the broader municipality, state, or nation. For example, the decision to adopt and implement a clean cookstove, such as a liquid purified gas (LPG) stove, in a household will be influenced not only by whether that cookstove is relatively simple to use, but also whether the household member(s) can access the gas required to power the stove or have access to technical assistance to use or repair the stove. The decision will also be based on cultural and behavioral factors in the home and community, which often drive adoption and acceptance. These frameworks guide our studies by informing our hypotheses about how interventions work, why they work, and what might impede or support the ability to implement them, which can inform a priori strategies to facilitate implementation.

The selection of frameworks will depend on the research questions and study objectives. In the clean cookstoves example, researchers used the RE-AIM Framework to understand the Reach, Effectiveness, Adoption, Implementation, and Maintenance of clean cookstoves across 11 low- and middle-income countries. This enabled them to identify key gaps in implementation and highlight areas for future efforts.

One important feature of implementation science frameworks is their attentiveness to multi-level factors that influence implementation, including characteristics of 1) the individuals delivering the intervention, 2) the organization in which it is delivered, and 3) the community in which those individuals and organization exist. This is critical for environmental health studies that seek to eliminate health disparities. Understanding contextual factors that influence equitable implementation can help us design and deliver interventions that will mitigate and not exacerbate existing environmental health disparities disproportionately affecting communities of color.

#### Implementation strategies

Implementation strategies are the focus of implementation science. They are defined as the “methods or techniques used to enhance the adoption, implementation and sustainability of an evidence-based program or practice” [17]. Most implementation studies seek to develop and test strategies to improve uptake and use of effective interventions. The purpose of these strategies is to improve the aforementioned proximal outcomes, such as the feasibility, adoption, or sustainability of an intervention [12]. Over 70 strategies have been classified into broad categories [17, 18], including evaluative and iterative strategies, interactive assistance, adapting and tailoring to context, developing stakeholder relationships, educating and training, engaging consumers, financial strategies, and strategies to change infrastructure. The selection of a strategy or set of strategies will depend on the implementation barriers being addressed and the implementers being targeted. For example, in efforts to overcome financial barriers for households to use LPG stoves, investigators tested conditional cash transfers as a financial incentive for adoption and use. In seeking to influence policymakers at the municipal, state, or national level for LPG stove distribution, studies might test strategies to develop stakeholder relationships and build buy in. Examples of these types of strategies include identifying champions, informing local opinion leaders, or building coalitions. If a major barrier is the ability to properly use and maintain the stove, studies might focus on strategies to provide interactive assistance, such as facilitation or technical assistance. Ultimately, implementation science seeks to understand which set of strategies work best in a particular context to improve implementation outcomes.

#### Study designs

Implementation science uses a variety of study designs typically used in other fields, including observational and experimental designs. Study designs that may be less familiar to environmental health researchers but that are commonly used in implementation science include quasi-experimental and effectiveness-implementation hybrid designs, as well as mixed methods designs [19]. The following paragraphs will review some of these designs in more detail and provide guiding examples.

##### Experimental designs

One popular experimental design is the stepped wedge design [20], which is a type of cluster randomized controlled trial (RCT). In the stepped wedge design an intervention is rolled out in multiple places (or clusters) sequentially rather than simultaneously, and comparisons can be made within and between clusters. The advantage of this design is that it is more feasible to focus resources in one place at a time. This can be particularly useful for a clean cookstove intervention trial that seeks to test strategies across multiple community settings but may not have the resources to implement the intervention across all settings at one time.

##### Quasi-experimental designs

While experimental designs test an intervention through randomization, quasi-experimental designs were developed to test interventions when randomization is not possible. This is often the case when pursuing questions related to mitigation or reduction of exposures to environmental pollutants. These include designs such as interrupted time series [21], regression discontinuity [22], and non-equivalent control group [23]. These designs are particularly useful in environmental health where it may be unethical to withhold an intervention that can prevent or reduce exposure harms. For example, in an interrupted time series (ITS) design everyone receives the intervention and multiple assessments are taken prior to and following the introduction of the intervention. An ITS design could be an appropriate design to study the impact and rollout of a lead abatement program which would be implemented broadly, including all eligible households in a community rather than withholding abatement from some participants. In selecting a study design, careful attention should be paid to the underlying assumptions, advantages, and disadvantages.

##### Hybrid designs

Effectiveness-implementation hybrid designs are another particularly useful design for environmental health researchers developing interventions. These designs have a dual focus a priori on assessing intervention effectiveness and implementation [24]. The overall goal is to accelerate the transition from effectiveness studies to implementation studies. There are three types of hybrid designs that vary by the emphasis placed on the aims of the study. At one end of the spectrum, Type 1 designs are primarily focused on studying intervention effectiveness while secondarily collecting information about implementation processes including barriers to implementation. For example, in developing an early warning system for disaster preparedness, researchers can test the effectiveness of the system in its ability to predict and monitor conditions allowing for communities to prepare for and adapt to disasters, while also studying how such a system would be implemented in a particular context (e.g., how would data be accessed, how would the system be run and maintained, how would output reach the affected communities, who would be ensuring these steps take place, etc.) At the other end of the spectrum, Type 3 designs are primarily focused on testing implementation strategies while secondarily collecting information on health outcomes. For example, researchers developed and tested an effective household intervention that increased the appropriate and exclusive use of clean cooking technologies through education, incentivization, and environmental restructuring [25, 26]. In a subsequent type 3 hybrid design, researchers could develop and test strategies that will support the ability to scale up the intervention across a range of settings while also assessing the intervention’s effectiveness to increase use of clean cookstoves, for example, through air monitoring to measure reductions in indoor air pollution. Type 2 designs fall in between Type 1 and Type 3 designs, with a dual primary focus of testing effectiveness and testing an implementation strategy. The type of hybrid design selected is dependent on the degree to which effectiveness of an intervention is already well characterized for a particular context. These designs offer an opportunity for environmental health researchers who are focusing on prevention to not only test the effectiveness of their interventions but also understand implementation processes required to deliver the intervention in a variety of settings. Understanding these processes and identifying the appropriate people to implement an intervention are the first steps in understanding factors that influence successful implementation, enabling subsequent implementation studies on strategies to increase the likelihood that these interventions will be effectively delivered in practice.

##### Mixed methods

Mixed methods designs are the collection and integration of qualitative and quantitative data to help understand processes and context. Where quantitative data can provide a measure of effect, qualitative data can reveal the reasons why and how things work or don’t work. For example, in a clean cookstove study that tests strategies to enhance LPG stove adoption and use, investigators may measure uptake and use by measuring particulate matter levels in household ambient air and may also use direct observation and/or conduct qualitative interviews and focus groups to understand how and why households used or did not use the LPG stoves. Understanding why a strategy to enhance implementation was effective or not allows for an iterative process to modify or adapt an intervention to fit a specific context, or to further refine a set of strategies to enhance implementation.

For environmental health researchers who have historically focused on documenting risks and understanding their causes, implementation science can further expedite efforts that shift the scientific focus towards better understanding *how* to promote prevention. Through focusing on the *how*, we can advance disease prevention and improve population health by understanding the most effective strategies that enable us to effectively communicate, integrate, and sustain interventions (including recommendations, guidelines, and policies) in practice. The following section enumerates ways that environmental health researchers can incorporate implementation science into their work at various stages of the translational research pathway, to bridge the gap from research to practice.

### Integrating implementation science into environmental health sciences

**Fig. 1:**
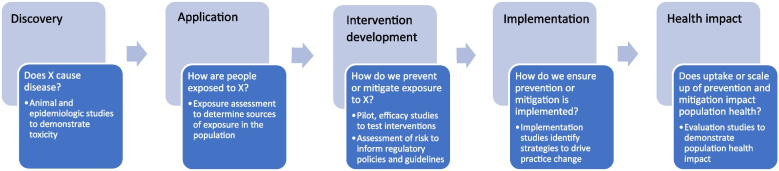
The Translational Pathway of Environmental Health Research from Discovery to Population Health Impact

Many have articulated the translational research pathway from basic discovery to human application to intervention development to implementation and evaluation [27–29]. However, these earlier models were developed primarily for medical research. More recently, the NIEHS developed the Translational Research Framework (https://www.niehs.nih.gov/research/programs/translational/framework-details/index.cfm) which serves as a guide for environmental health scientists to envision how their studies can move along this pathway. Importantly, this framework highlights the development of evidence-based interventions and implementation science playing key roles in this iterative (and not always linear) process [30]. Figure 1 illustrates the translational research pathway in the context of environmental health research, elaborating on the role of implementation science.

Much of environmental health research has focused on the first stages of this pathway, from basic discovery to application to intervention development, namely, informing recommendations, guidelines, and regulatory policies, as well as health interventions. For example, environmental health researchers have made substantial progress in basic discovery by identifying and characterizing chemical toxicity through in vitro, in silico, animal, and epidemiologic studies. These discoveries have led to research that focuses on the underlying mechanisms by which these exposures impact biology and pathophysiology and research on understanding how people are exposed to these chemicals across the lifespan in multiple environments. Through exposure assessments, environmental health researchers have elucidated the relevant chemical species and main sources of exposure. This application then leads to science that would inform the development of policies, recommendation, and guidelines, such as risk assessments that would reduce exposures to a safe level in a population. Further, understanding the relevant sources of exposure can inform prevention or mitigation efforts. Thus, interventions can be developed that can prevent or mitigate human exposure to these relevant sources. For example, to reduce exposure to household air pollution, a variety of clean cooking technologies have been developed for households with unreliable access to electricity or gas. However, these clean cookstove technologies are not necessarily being adopted nor used appropriately by households [31]. Once interventions are developed and tested, implementation studies can inform the most effective strategies to ensure their adoption and appropriate use, and ultimately, how best to scale up these interventions to all populations who can benefit. In the example of cookstoves, the Clean Cooking Implementation Science Network [31] has studied a range of strategies to promote adoption and appropriate use, including strategies to increase awareness [32] and financial strategies to incentivize replacement of polluting stoves [33]. Finally, evaluation studies can assess the health impact of these implementation and scale up efforts [34].

Implementation science has evolved and developed methods not only to focus on the later stages of the translational research pathway, but also to inform intervention development studies. In studying human application and intervention development, researchers should consider who will be delivering the intervention and how it fits with the ultimate consumer population, and build in tests of training, support, and adherence. For example, effectiveness-implementation hybrid designs, whereby researchers can dually study intervention effectiveness as well as implementation [24], as described above in the examples of early warning systems and clean cookstoves, can help to speed up the translational research process by integrating implementation studies and intervention development studies. By designing interventions for dissemination and implementation, researchers can increase the likelihood that the outputs of their research will be implemented in practice. For example, the Household Air Pollution Intervention Network Trial is a multi-country trial testing the effectiveness of clean cooking technologies on improving health outcomes [35] while also studying approaches to improve use of the clean cookstoves [36].

We have a tremendous opportunity to use implementation science to understand how environmental health evidence is being disseminated, implemented, and sustained, and how best to address the gaps in those processes. Institutes, centers, and offices across the NIH recognize the importance of advancing our understanding of the most effective strategies to integrate evidence-based interventions within community, clinical, and public health systems. Thus, they have issued a set of funding announcements in Dissemination and Implementation Research in Health (PAR-22-105, PAR-22-106, PAR-22-109), which seek to build the knowledge base on how to effectively disseminate, implement, sustain, and scale evidence-based interventions, as described in the previous section. These institutes and centers include several focused on environmental exposures, including NIEHS, National Cancer Institute (NCI), *Eunice Kennedy Shriver* National Institute of Child Health and Human Development, National Institute of Mental Health, National Heart, Lung, and Blood Institute, and Fogarty International Center. At NIEHS, the integration of implementation science is developing throughout the portfolio, including a new initiative on children’s health that supports collaborations between environmental health scientists and implementation scientists (https://grants.nih.gov/grants/guide/rfa-files/rfa-es-20-001.html). NIEHS supported scientists are also involved in the NIH-wide RADxUP program which is conducting implementation science related to the uptake of COVID19 testing in communities experiencing health disparities and environmental injustice.

In addition to funding opportunities, NIH supports a range of training opportunities. For example, the NCI hosts the Training Institute in Dissemination and Implementation Research in Cancer (TIDIRC), which provides thorough instruction in conducting dissemination and implementation studies and is now available in open access format (https://cancercontrol.cancer.gov/IS/training-education/tidirc/openaccess.html). Additional implementation science resources for environmental health researchers can be found at the NIEHS website (https://www.niehs.nih.gov/research/supported/translational/implementation/index.cfm) and the NCI Implementation Science Team website (https://cancercontrol.cancer.gov/IS/).

## Conclusions

Environmental health researchers can advance disease prevention not only by informing the development of effective interventions, regulatory policies, and guidelines, but also by ensuring that those interventions, policies, and guidelines are effectively adopted, implemented and sustained. They can do this by incorporating implementation science into their work across the translational research pathway. Namely, environmental health researchers can design interventions that better fit the context in which they are meant to be applied. Further, they can study implementation processes and inform implementation strategies to most effectively implement those interventions. A variety of methods and study designs in implementation science have been described in this paper that can guide environmental health researchers to conduct implementation studies. By fully integrating the concepts, methods, and findings of implementation science into the environmental health research agenda, we can envision a more comprehensive flow from research to practice that maximizes the use of scientific discovery and supports the NIEHS mission of discovering how the environment affects people in order to promote healthier lives.
